# Tricyclic antidepressants for major depressive disorder: a comprehensive evaluation of current practice in the Netherlands

**DOI:** 10.1186/s12888-021-03490-x

**Published:** 2021-10-01

**Authors:** Cornelis F. Vos, Rob E. Aarnoutse, Marijke J. M. Op de Coul, Jan Spijker, Mascha M. Groothedde-Kuyvenhoven, Raluca Mihaescu, Sonja J. W. Wessels-Basten, Jordy J. E. Rovers, Sophie E. ter Hark, Aart H. Schene, Marlies E. J. L. Hulscher, Joost G. E. Janzing

**Affiliations:** 1 Department of Psychiatry, Radboud University Medical Center, Reinier Postlaan 10, 6500 HB Nijmegen, The Netherlands; 2 Donders Institute for Brain, Cognition and Behaviour, Radboud University, Nijmegen, The Netherlands; 3 Department of Pharmacy, Radboud Institute for Health Sciences, Radboud University Medical Center, Nijmegen, The Netherlands; 4 Radboud University, Nijmegen, The Netherlands; 5 Pro Persona, Nijmegen, The Netherlands; 6 Deventer Hospital, Deventer, The Netherlands; 7 Dimence Group, Deventer, The Netherlands; 8 Catharina Hospital Eindhoven, Eindhoven, The Netherlands; 9 Vincent van Gogh Institute for Psychiatry, Venray, The Netherlands; 10 Scientific Center for Quality of Healthcare, Radboud University Medical Center, Nijmegen, The Netherlands

**Keywords:** Antidepressant agents, Clinical practice guidelines, Qualitative approach, Major depressive disorder, Tricyclic antidepressants

## Abstract

**Background:**

Traditionally tricyclic antidepressants (TCAs) have an important place in treatment of major depressive disorder (MDD). Today, often other antidepressant medications are considered as first step in the pharmacological treatment of MDD, mainly because they are associated with less adverse effects, whereby the position of TCAs appears unclear. In this study we aimed to examine the current practice of TCAs in treatment of unipolar MDD.

**Methods:**

A mixed methods approach was applied. First, a selection of leading international and national guidelines was reviewed. Second, actual TCA prescription was examined by analyzing health records of 75 MDD patients treated with the TCAs nortriptyline, clomipramine or imipramine in different centers in the Netherlands. Third, promotors and barriers influencing the choice for TCAs and dosing strategies were explored using semi-structured interviews with 24 Dutch psychiatrists.

**Results:**

Clinical practice guidelines were sometimes indirective and inconsistent with each other. Health records revealed that most patients (71%) attained therapeutic plasma concentrations within two months of TCA use. Patients who achieved therapeutic plasma concentrations reached them on average after 19.6 days (*SD* 10.9). Both health records and interviews indicated that therapeutic nortriptyline concentrations were attained faster compared to other TCAs. Various factors were identified influencing the choice for TCAs and dosing by psychiatrists.

**Conclusions:**

Guideline recommendations and clinical practice regarding TCA prescription for MDD vary. To increase consistency in clinical practice we recommend development of an up-to-date guideline integrating selection and dosing of TCAs, including the roles of therapeutic drug monitoring and pharmacogenetics. Such a guideline is currently lacking and would contribute to optimal TCA treatment, whereby efficacy and tolerability may be increased.

**Supplementary Information:**

The online version contains supplementary material available at 10.1186/s12888-021-03490-x.

## Introduction

Major depressive disorder (MDD) is among the leading causes of disability worldwide with a lifetime risk of 15 to 18% and over 264 million people affected globally [1, 2]. MDD is commonly treated with antidepressant medications, such as tricyclic antidepressants (TCAs), selective serotonin reuptake inhibitors (SSRIs) and serotonin-noradrenaline reuptake inhibitors (SNRIs). TCAs have been of foremost importance in pharmacotherapy of MDD since their introduction in the 1950s [3]. However, over the last decades, SSRIs, SNRIs and other modern antidepressants are increasingly considered as first-line agents, mainly because they are associated with less adverse effects and since they are less dangerous in overdose [3–6]. TCAs continue to be prescribed, if only because of the common occurrence of non-response to SSRIs and SNRIs, but the current position of TCAs in pharmacotherapy of unipolar MDD is unclear [3, 4].

An advantage of TCAs over SSRIs and SNRIs is that therapeutic plasma concentrations associated with optimal efficacy are defined [7]. Subtherapeutic concentrations are related to suboptimal or non-response whereas supratherapeutic concentrations are associated with higher rates of adverse effects. Importantly, there are considerable interindividual differences between TCA doses administered and plasma concentrations achieved, notably because of variation in activity of the cytochrome P-450 (CYP) enzymes by which TCAs are metabolized [8]. During TCA treatment plasma concentrations can be monitored to guide dosing until an adequate maintenance dose is reached (therapeutic drug monitoring; TDM) [7].

So far, it has not been investigated what factors influence the choice for TCAs instead of other antidepressants by psychiatrists. As a result, the present-day significance of TCAs in pharmacotherapy of MDD remains unclarified. Also, it is unknown how long it takes until therapeutic TCA plasma concentrations are attained, which impairs the ability to determine to what extent novel dosing strategies, such as genotype-informed dosing, may accelerate TCA dose-adjustment [8, 9].

The aim of this study was to examine the current practice of TCA treatment for adult patients with unipolar MDD. Our objectives were to assess guideline recommendations on TCA treatment, to investigate real-world TCA prescription and to gain understanding of underlying factors influencing TCA selection and dosing by psychiatrists. It was hypothesized that TCA prescription was highly influenced by individual preferences of psychiatrists, rather than a straightforward application of the guidelines. This would be problematic, since variation in antidepressant prescription suggests suboptimal treatment for at least a subgroup of patients.

## Methods

### Study design

Since we aimed to investigate guideline recommendations, actual TCA treatment and factors influencing TCA prescription by psychiatrists, a mixed methods approach was used. First, recommendations from antidepressant prescribing guidelines were examined because it was assumed that these were leading for TCA prescription in clinical practice. Second, health records were analysed of MDD patients treated with TCAs to examine actual TCA prescription. Third, promotors and barriers influencing TCA treatment were explored by conducting semi-structured interviews with psychiatrists.

In this study, the TCAs nortriptyline, clomipramine, imipramine and amitriptyline were investigated, as these are commonly prescribed and recommended in treatment of MDD in the Netherlands [10]. Amitriptyline was not included in the analysis of health records since particularly this TCA is often prescribed for other indications, such as neuropathic pain, which could be difficult to distinguish in health records [11].

### Guideline recommendations

To provide an overview of guideline directions two leading European, two North-American, two international (global) and the Dutch national guideline on treatment of MDD were reviewed, as presented in Table 1. Guidelines were selected considering their relevance for TCA prescription in a Dutch setting with the consensus of all authors.

**Table 1:** Selected clinical practice guidelines on the pharmacological treatment of unipolar MDD

Guideline	Country
Depression in Adults: Recognition and Management, by the National Institute for Health and Care Excellence (NICE), 2020 [12]	England
Unipolare Depression, by the Deutsche Gesellschaft für Psychiatrie, Psychotherapie und Nervenheilkunde (DGPPN), 2015 [13]	Germany
Practice Guideline for the Treatment of Patients with Major Depressive Disorder, by the American Psychiatric Association (APA), 2010 [14]	United States
Clinical Guidelines for the Treatment of Major Depressive Disorder, by the Canadian Network for Mood and Anxiety Treatments (CANMAT), 2016 [15]	Canada
Antidepressants in Treatment of Adults with Depression, by the World Health Organization (WHO), 2012 [16]	Global
World Federation of Societies of Biological Psychiatry (WFSBP) Guidelines for Biological Treatment of Unipolar Depressive Disorder, 2013 [17]	Global
Multidisciplinary Guideline for Treatment of Depressive Disorders, by the Trimbos Institute, 2013 [10]; including the Addendum for Treatment of Elderly Patients, Trimbos Institute, 2008 [18]	The Netherlands

In addition, summaries of product characteristics (SmPC) of nortriptyline, clomipramine, imipramine and amitriptyline were reviewed. In absence of SmPCs by the European Medicines Agency, we only reviewed those approved by the Dutch Medicines Evaluation Board [19–22] and the United States Food and Drug Administration [23–26]. Furthermore, international consensus guidelines on TDM and pharmacogenetics were selected and reviewed [7, 8].

### Health records

To investigate actual TCA prescription, 75 health records were analyzed of MDD patients treated with nortriptyline, clomipramine or imipramine divided over five centers in the Netherlands. The centers included one academic hospital (Radboudumc, Nijmegen), one general hospital (Catharina Hospital, Eindhoven) and three large psychiatric institutions (Pro Persona, Nijmegen; Vincent van Gogh, Venray; Dimence Group, Deventer). Permission was obtained from ethical review boards or boards of directors for each center.

In each center 15 records were selected randomly. First, all adult patients treated with nortriptyline, clomipramine or imipramine between January 2014 and June 2018 were extracted from the electronic institutional system. Second, records were selected in the order of a random numbers list generated in SPSS version 26 (IBM Corp, Armonk, NY) until 15 suitable records were selected per center. Inclusion criteria were treatment with nortriptyline, clomipramine or imipramine for unipolar MDD, diagnosed according to DSM-IV-TR or DSM-5 criteria [27, 28], and TCA use for at least four consecutive weeks. An exclusion criterium was participation in scientific research influencing TCA prescription. Dosing was analyzed during the first 2 months of TCA use. Selection and dosing characteristics were compared between the TCAs using *X*^2^-tests or one-way ANOVA where appropriate.

### Semi-structured interviews

Semi-structured interviews were conducted to identify factors influencing TCA prescription in clinical practice. An interview guide was developed by the authors and based on the model of Flottorp et al. [29] (Supplement 1). Psychiatrists were selected varying in gender, years of professional experience, center (a maximum of two psychiatrists per center), type of center (academic hospital, general hospital, general psychiatric institution or private practice holder), setting (hospitalized versus outpatient) and geographical location in the Netherlands. An inclusion criterium was TCA prescription for MDD at least once per 3 months over the last year.

Interviews were held face-to-face by two interviewers (CV and MOdC), either on location or by videocall, between July and September 2020. Permission for audio-recording and use of data was obtained from all psychiatrists. Interviews were held until no new information was identified. Subsequently, interviews were transcribed verbatim and coded and analyzed in Atlas by both interviewers (Atlas.ti 8.4.20, Windows).

## Results

### Guideline recommendations on TCA selection

To examine guideline directions on TCA prescription, selected antidepressant prescribing guidelines and TCA product characteristics were reviewed. Clinical practice guideline recommendations on TCA selection are summarized in Table 2. All reviewed guidelines consider the overall efficacy of TCAs, SSRIs and SNRIs comparable. Also, they agree that TCAs are associated with more adverse effects and that they are more dangerous in overdose. Therefore, the National Institute for Health and Care Excellence (NICE), American Psychiatric Association (APA) and Canadian Network for Mood and Anxiety Treatments (CANMAT) recommend TCAs as second- or third-line treatment options [12, 14, 15].

**Table 2:** Clinical guideline recommendations on TCA selection in pharmacotherapy of unipolar MDD

Institute, year	Position of TCAs^§^	Preference between NOR, CLO, IMI and AMI^†^
NICE, 2020 [12]	First-line: SSRI. First switch to another modern antidepressant prior to venlafaxine, TCA or monoamine oxidase inhibitor.	No preference.
DGPPN, 2015 [13]	First-line options include SSRIs, SNRIs and TCAs. Comorbid diabetes mellitus: no sedative TCA. Comorbid pain syndrome: TCA.	NOR: least adverse effects. Avoid CLO in combination with monoamine oxidase inhibitors.
APA, 2010 [14]	First-line: SSRI SNRI, mirtazapine or bupropion. Second-line treatment options include TCAs.	NOR: elderly patients, Parkinson’s disease, smoking cessation.
CANMAT, 2016 [15]	First-line: SSRI, SNRI, mirtazapine, bupropion, agomelatine, mianserin, milnacipran, vortioxetine. Second-line options include TCAs.	CLO: MDD with anxious distress.
WHO, 2012 [16]	First-line options include SSRIs, SNRIs and TCAs. Elderly patients: SSRI. During pregnancy: TCA or fluoxetine.	No preference.
WFSBP, 2013 [17]	First-line: SSRI, bupropion or mirtazapine in mild depression and SSRI, SNRI or TCA in severe depression.	NOR: least adverse effects, e.g., in elderly patients. CLO: comorbid OCD^¶^.
Dutch Multidisciplinary Guideline, 2013, 2008 [10, 18]	First-line: SSRI, SNRI, mirtazapine or bupropion. Hospitalized patients: TCA. Elderly patients: SSRI. Psychotic depression: TCA.	NOR: elderly patients. CLO: comorbid anxiety disorder, OCD.

^§^ Position of TCAs relative to other first- or second-line options in the pharmacological treatment of unipolar major depressive disorder (MDD)

^†^ NOR = nortriptyline, CLO = clomipramine, IMI = imipramine, AMI = amitriptyline

^¶^ Obsessive-compulsive disorder

Guidelines by the World Health Organization (WHO), World Federation of Societies of Biological Psychiatry (WFSBP), Deutsche Gesellschaft für Psychiatrie, Psychotherapie und Nervenheilkunde (DGPPN) and the Dutch guideline state no general preference between TCAs, SSRIs, SNRIs and the other modern antidepressants mirtazapine and bupropion, except for subgroups of patients [10, 13, 16–18]. The WHO advises TCAs or the SSRI fluoxetine during pregnancy [16], the DGPPN advises TCAs in patients with comorbid pain syndrome [13] and the Dutch guideline recommends TCAs in psychotic depression and in hospitalized MDD patients [10, 18]. Concerning elderly patients, the WHO, WFSBP and Dutch guideline prefer SSRIs over TCAs [16–18]. If a TCA is selected in elderly patients, generally nortriptyline is preferred as it is associated with the least adverse effects among the TCAs [13, 14, 17, 18]. Several guidelines recommend clomipramine in patients with a comorbid anxiety disorder or obsessive-compulsive disorder (OCD) because of its serotonergic profile [10, 15, 17].

Apart from SSRIs, SNRIs, TCAs, mirtazapine and bupropion, first- and second-line treatment options may include other antidepressant medications, such as multi-modal antidepressants which combine different modes of action, e.g. vortioxetine and agomelatine [15, 30]. Also, a number of guidelines consider combining antidepressants or augmentation with other psychopharmaceuticals, in particular antipsychotics, as second-line treatment option.

Summaries of TCA product characteristics highlight contra-indications for TCAs, particularly recent myocardial infarction and co-use of monoamine oxidase (MAO) inhibitors [19–26]. The reviewed documents on TDM and pharmacogenetics do not consider TCA selection [7, 8].

### Guideline recommendations on TCA dosing

Directions on TCA dosing are presented in Table 3. All selected guidelines advise to start with a lower, non-therapeutic dosage to minimize adverse effects and to prevent early discontinuation. Subsequently, the dose should be increased gradually until an adequate maintenance dose is reached.

**Table 3:** Recommendations on TCA dosing in treatment of unipolar MDD

	Nortriptyline	Clomipramine	Imipramine	Amitriptyline
Initial dose (mg/day)	25^a^, 25-50^b,c^ or 50-75^d^	25-50^b,c^ or 50-75^f^	25-50^b,c^	25-50^a,b,c^ or 50^h^
	EP^†^: 10-30^d^ or 12.5-25^b^	EP: 10^f^ or 12.5-25^b^	Outpatient: 25-75^g^	CVD^‡^: 10-25^h^
			Hospitalized: 75-100^g^	EP: 10-25^h^ or 12.5-25^b^
			EP: 10^g^ or 12.5-25^b^	
Maintenance dose (mg/day)	50-200^a,b^, 75-200^c^	75-250^b^, 100-150^c^	75-300^f^ or 100-300^a,c^	75-300^b^, 100-300^a^
	or 100-150^d^	or max. 250^f^	Outpatient: 150-200^g^	or max. 150^h^
	Hospitalized: 100-200^d^	EP: 30-50^f^	Hospitalized: 200-300^g^	
	EP: 50-150^d^		EP: 20-50^g^	
Therapeutic plasma	50-150^d^ or 70-170^b,c,e^	175–450^§,c^ or	175–300^§,b,c,e^	80–200^§,b,c,e^
concentrations (ng/mL)		230–450^§,b,e^		

^†^ Elderly patients; ^‡^ Cardiovascular disease; ^§^ Sum of TCA and active metabolite

^a^ American Psychiatric Association, 2010 [14]; ^b^ DGPPN, 2015 [13]; ^c^ WFSBP, 2013 [17]; ^d^ Mallinckrodt Pharmaceuticals, 2012 [23]; ^e^ Hiemke et al., 2018 [7]; ^f^ Mallinckrodt Pharmaceuticals, 2014 [24]; ^g^ Excellium Pharmaceuticals Inc., 2012 [25]; ^h^ Sandoz, 2014 [26]

The NICE guideline recommends a lower initial TCA dosage in case of switching from fluoxetine or paroxetine since these SSRIs influence TCA plasma concentrations [12]. Also, the NICE advises to maintain and no longer increase the TCA dosage if treatment response occurs [12]. All guidelines agree that dosing in elderly patients should be more gradual. Furthermore, hospitalization and cardiovascular disease may affect the dosing strategy, as shown in Table 3.

Most guidelines advise TDM in case of non-response [10, 13, 14, 18]. The DGPPN strongly recommends TDM during TCA up-titration in general and particularly in patients with non-response, adverse effects, if the maximum dose is used, if comedications are prescribed or in case of comorbidities [13]. The Dutch guideline advises TDM in case of non-response and in elderly patients with severe depression [10, 18].

TCA metabolization is influenced by activity of relevant cytochrome P-450 (CYP) enzymes, depending on the patients' genotype, which results in interindividual differences in the relation between TCA dose administered and plasma concentration achieved [8]. For poor, intermediate and ultra-rapid metabolizers of CYP2D6 and CYP2C19, genotype-informed dosing recommendations are available [8]. Several selected documents consider pharmacogenetics to personalize TCA dosing but none indicates explicitly when pharmacogenetic testing should be applied. The CANMAT dissuades routine pharmacogenetic testing [15].

### TCA prescription in clinical practice

To investigate TCA prescription in clinical practice, 75 health records were examined of MDD patients treated with nortriptyline (*n* = 53, 71%), clomipramine (*n* = 18, 24%) or imipramine (*n* = 4, 5%), as presented in Table 4. Most patients older than 65 years were treated with nortriptyline. Patients with comorbid anxiety, defined as MDD with anxious distress or MDD with a comorbid anxiety disorder, were treated with nortriptyline (*n* = 5) or clomipramine (*n* = 2).

**Table 4:** Analysis of health records of MDD patients treated with nortriptyline, clomipramine or imipramine (*n* = 75)

	Total (*n* = 75)	Nortriptyline (*n* = 53)	Clomipramine (*n* = 18)	Imipramine (*n* = 4)	*p*-value^†^
Patient characteristics					
Female, *n* (%)	39 (52)	27 (51)	9 (50)	3 (75)	0.64
Age, mean (*SD*)	54.4 (16.6)	54.5 (17.1)	52.6 (15.1)	60.8 (18.9)	0.21
Hospitalized, *n* (%)	44 (59)	33 (62)	9 (50)	2 (50)	0.62
Psychotic depression, *n* (%)	16 (21)	12 (23)	4 (22)	0 (0)	0.56
TCA selection					
Age > 65 years, *n* (%)	21 (28)	17 (32)	3 (17)	1 (25)	0.44
Comorbid anxiety^‡^, *n* (%)	7 (9)	5 (9)	2 (11)	0 (0)	0.79
TCA dosing					
Initial dose (mg/day), mean (*SD*)	40.3 (19.5)	35.2 (14.1)	58.2 (23.4)	27.5 (16.6)	0.001**
Max. dose^§^ (mg/day), mean (*SD*)	105.1 (39.6)	90.3 (31.1)	133.3 (32.4)	175.0 (28.9)	< 0.001**
TDM conducted^§^, *n* (%)	67 (89)	46 (87)	17 (94)	4 (100)	0.51
Therapeutic concentrations^§^, *n* (%)	53 (71)	42 (79)	9 (50)	2 (50)	0.040*
Pharmacogenetics^¶^, *n* (%)	5 (7)	5 (9)	0 (0)	0 (0)	0.33

* *p* = ≤0.05, ** *p* = ≤0.001 (two-tailed); ^†^ One-way ANOVA was used to compare age, initial and maximum dose. *X*^2^-tests were used to compare the other variables; ^‡^ MDD with anxious distress (*n* = 1) or comorbid anxiety disorder (*n* = 6) [27, 28]; ^§^ Within two months since TCA initiation; ^¶^ Applied to optimize TCA dosing

Dosing was analyzed regarding the first 2 months of TCA use. Initial and maximum doses differed between the TCAs in accordance with guideline recommendations (see Table 3). Pharmacogenetics was applied in five patients (7%) to personalize TCA dosing. Within the first 2 months of treatment, TDM was applied in 67 patients (89%). In total, 53 patients (71%) attained therapeutic plasma concentrations, on average after 19.6 days (*SD* 10.9). For nortriptyline, 42 patients (79%) reached therapeutic plasma concentrations, averagely after 17.2 days (*SD* 8.1). Nine patients using clomipramine (50%) attained therapeutic plasma concentrations, on average after 25.6 days (*SD* 14.9) and two patients treated with imipramine (50%) reached therapeutic plasma concentrations, on average after 43.5 days (*SD* 0.7).

The number of patients attaining therapeutic plasma concentrations differed significantly between the TCAs, *X*^2^ (1, *n* = 75) = 6.415, *p* = 0.040. Considering the 67 patients for whom TDM was applied, patients treated with nortriptyline reached therapeutic plasma concentrations more frequently compared to patients using either clomipramine or imipramine, *X*^2^ (1, *n* = 67) = 6.415, *p* = 0.011. Regarding the 53 patients attaining therapeutic plasma concentrations, the number of days until these were reached differed significantly between the TCAs: one-way ANOVA, *F* (24,28) = 3.619, *p* = 0.001.

### Promotors and barriers regarding TCA prescription

Semi-structured interviews were conducted with 24 psychiatrists. Their demographics are presented in Table 5. Interviews lasted at least half an hour per interview. Four psychiatrists treated elderly patients exclusively.

**Table 5:** Demographics of interviewed psychiatrists (*n* = 24)

Demographic	Psychiatrists (*n* = 24)
Female, *n* (%)	10 (42)
Age, mean (range)	50.1 (34–68) years
Years of experience as psychiatrist, mean (range)	16.3 (1–38) years
Type of center	
Academic hospital, *n*	4
General hospital, *n*	3
General psychiatric institution, *n*	14
Private practice holder, *n*	3
Type of patients	
Hospitalized, *n*	9
Outpatients, *n*	15

#### Factors influencing TCA selection

**Fig. 1:**
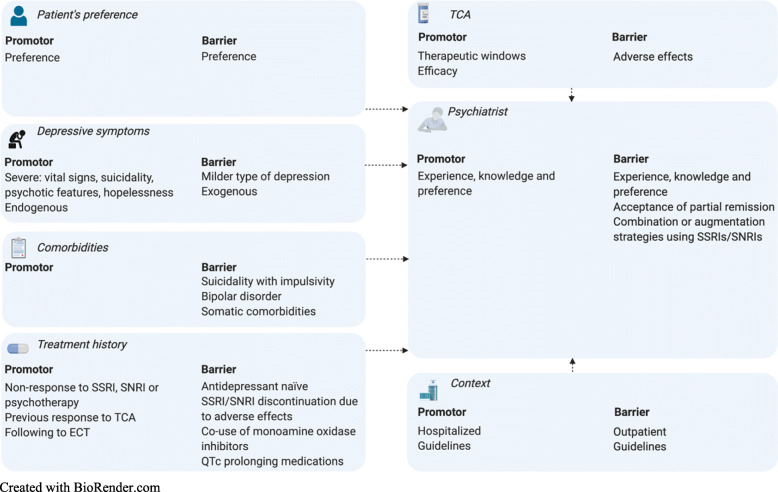
Most important factors influencing TCA selection by psychiatrists

Factors influencing TCA selection by psychiatrists were divided into several categories as presented in Fig. 1: patient’s preference, depressive symptoms, comorbidities, treatment history, TCA related factors, psychiatrist related factors and contextual factors.

##### Patient’s preference

All interviewed psychiatrists mentioned the importance of shared decision making: “It is partly what you know from literature, but it is also making a story together what is acceptable for both”.

##### Depressive symptoms

All psychiatrists were more inclined to prescribe TCAs for severe, rather than for a milder type of depression. Severe depression was described as depression with vital signs, suicidality, psychotic or catatonic features, psychomotor retardation or hopelessness. Most were more tempted to prescribe TCAs in case of an endogenous, biological profile of depression, characterized by recurrent episodes or a family history of depression. In case of a more exogeneous profile, for instance if psychosocial factors contributed significantly, psychotherapy was favored. Many psychiatrists selected nortriptyline, instead of other TCAs, if tiredness was a major symptom of depression. Amitriptyline was preferred in agitated depression or in case of prominent insomnia.

##### Comorbidities

Comorbidities could hinder TCA selection in clinical practice. Relevant psychiatric comorbidities were bipolar disorder, a personality structure causing or maintaining depression, which favored psychotherapy, and manifest suicidality combined with impulsivity, due to the risk of TCA auto-intoxications. Comorbid anxiety disorder, OCD and obsessive-compulsive personality disorder promoted clomipramine instead of other TCAs. Relevant somatic comorbidities were prolonged QT interval, cardiac disease, hypotension, Parkinson’s disease, neurocognitive disorders and a history of a myocardial infarction. Among the TCAs, nortriptyline was preferred in case of relevant somatic comorbidities and in elderly patients. Amitriptyline or nortriptyline was generally prescribed in patients with comorbid pain syndrome.

##### Treatment history

None of the psychiatrists preferred TCAs as first-line treatment in the pharmacological treatment of antidepressant-naïve patients with non-psychotic MDD, both regarding hospitalized and outpatients. Non-response to SSRIs, SNRIs and psychotherapy promoted the choice for TCAs. TCAs were also more frequently prescribed if they proved effective in a previous depressive episode or directly following to electroconvulsive therapy (ECT). Discontinuation of SSRIs or SNRIs due to adverse effects promoted a different SSRI or SNRI. Also, use of monoamine oxidase (MAO) inhibitors or QTc prolonging co-medications hindered TCA selection.

##### TCA related factors

A promotor for TCAs was that therapeutic windows are defined, enabling psychiatrists to dose on the basis of plasma concentrations. Many psychiatrists confirmed that most patients experience more adverse effects to TCAs than to SSRIs. A barrier for amitriptyline and promotor for nortriptyline was that driving in the Netherlands is not allowed using amitriptyline, but is allowed using nortriptyline after the first week of treatment.

##### Psychiatrist related factors

According to the interviewed psychiatrists, the choice for an antidepressant is influenced by training, experience, personal preference and clinical setting. Some mentioned that psychiatrists may have less experience with TCAs compared to modern antidepressants, possibly resulting in restraint to select them: “They only know the results of epidemiological studies, not the pharmacological characteristics. They can’t personalize their treatment”. Others suggested that psychiatrists may accept partial remission in treatment with SSRIs: “It’s one of the biggest pitfalls: acceptance of partial remission. And it happens”. Furthermore, psychiatrists may combine antidepressants or augment antipsychotics prior to switching to TCAs.

##### Contextual factors

Hospitalization facilitated TCA selection. Hospital psychiatrists prescribed TCAs more frequently, especially compared to private practice holders. Several psychiatrists mentioned that the opinion of colleagues influenced their antidepressant selection. Many used the Dutch Multidisciplinary guideline on treatment of depressive disorders [10]. Some worked with local, center-specific guidelines.

#### Factors influencing TCA dosing

**Fig. 2:**
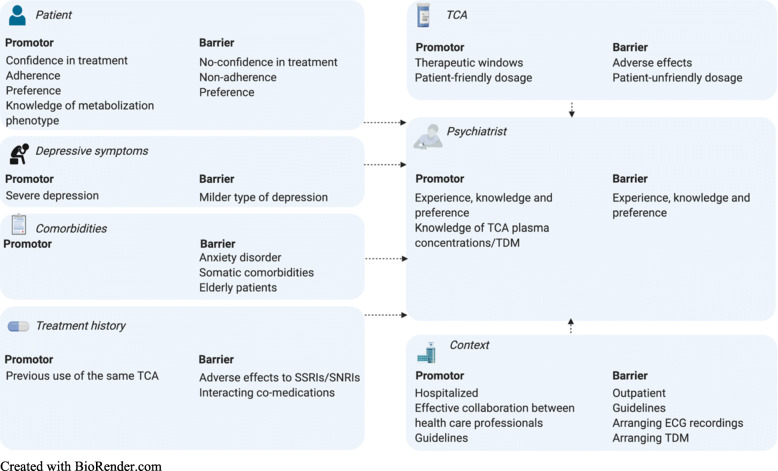
Most important factors influencing TCA dose-adjustment by psychiatrists

Factors influencing TCA dosing were subdivided in similar categories, as presented in Fig. 2: patient related factors, depressive symptoms, comorbidities, treatment history, TCA related factors, psychiatrist related factors and contextual factors. TCA dosing comprises prescription of the initial dosage and subsequent dose-adjustment. In the interview optimal dose-adjustment was defined as a limited amount of time until the maintenance dose is attained combined with exposing the patient to a minimum of adverse effects.

##### Patient related factors

Most psychiatrists decided on dosing together with the patient, which was thought to promote confidence in treatment, adherence and treatment success. Only one psychiatrist conducted pharmacogenetic testing regularly to optimize TCA dosing. However, if available most psychiatrists would take the metabolization phenotype into account.

##### Depression and comorbidities

Depression severity did not influence initial TCA dosing, although severe depression incited faster dose-adjustment, particularly in hospitalized patients. Some psychiatrists experienced that patients may discontinue treatment during rapid TCA up-titration and were more cautious in dose-adjustment. Most psychiatrists dosed more gradually in patients with comorbid anxiety disorder, as TCAs may luxate anxiety, in patients with somatic comorbidities and in elderly patients. Several psychiatrists specifically emphasized the importance of adequate dose-adjustment in elderly patients as they perceived that TCA dosages were often not up-titrated until therapeutic plasma concentrations were reached: “Start low, then go, but go all the way”.

##### Treatment related factors

Previous TCA use promoted faster dose-adjustment, especially if the required dose was known. Most psychiatrists dosed more gradually if patients experienced a lot of adverse effects to previous antidepressants or if they used co-medications causing potential drug-drug interactions with the prescribed TCA.

The majority of interviewed psychiatrists perceived problems in attaining therapeutic plasma concentrations, although significantly less for nortriptyline compared to other TCAs. According to many psychiatrists, usual nortriptyline maintenance doses often correspond with therapeutic plasma concentrations, while usual clomipramine, imipramine or amitriptyline maintenance doses frequently result in subtherapeutic plasma concentrations. Hence, especially for clomipramine, imipramine and amitriptyline, multiple dose-adjustments were often necessary to attain therapeutic plasma concentrations.

A barrier concerning imipramine was that tablets of max 25 mg are available in the Netherlands requiring patients to use many tablets per day, which was thought to hinder adherence.

##### Psychiatrist related factors

According to the interviewed psychiatrists, dosing was influenced by training, knowledge and experience. Most psychiatrists applied TDM within the first 2 weeks of treatment: “I always monitor plasma concentrations, since the initial dosage does mostly not result in therapeutic plasma concentrations”. Some increased the dose in expectation of the result: “I measure plasma concentrations and increase the dose directly. If plasma concentrations are too high, I decrease the dose afterwards”. Others measured plasma concentrations less frequently: “I dose on the basis of the clinical picture. Measuring plasma concentrations is rather out of frustration”.

##### Contextual factors

Many psychiatrists highlighted the importance of effective collaboration with other health care professionals. Several perceived arranging ECG recordings and TDM as time consuming, depending on the local situation. According to multiple psychiatrists, an outpatient setting could hinder dose-adjustment as psychiatrists see outpatients less frequently. For TCA dosing, most psychiatrists used the Dutch Multidisciplinary Guideline on treatment of depression [10, 18] and the online Dutch Pharmacotherapeutic Compass [31]. Several psychiatrists mentioned that concise instructions concerning TCA up-titration, TDM and pharmacogenetics were lacking, potentially leading to inconsistencies in clinical practice. Some worked with local, center-specific guidelines.

## Discussion

### Main study results

In this study we present a comprehensive evaluation of TCA prescription for MDD, focusing on Dutch psychiatrists. Our evaluation of selected guidelines demonstrated that only for subgroups of MDD patients is specified whether a TCA should be prescribed. Also, if a TCA is recommended, only for subgroups of patients is specified which TCA should be prescribed. Current practice guidelines provided little direction on how TCAs should be up-titrated in an effective, tolerable way, and what role TDM and pharmacogenetics should play herein. Our findings are in line with several reviews on antidepressant prescribing guidelines, reporting that these are partially indirective and inconsistent with each other, which can be partially accounted for by limited evidence from clinical trials [32–34].

Health records indicated that Dutch psychiatrists preferred nortriptyline over other TCAs in treatment of MDD. Most analyzed patients reached therapeutic plasma concentrations within 2 months of TCA use. Patients who attained therapeutic plasma concentrations reached them on average after 19.6 days. Time until attainment of therapeutic TCA plasma concentrations in a naturalistic setting has not been reported previously. Patients treated with nortriptyline reached therapeutic plasma concentrations faster compared to patients using clomipramine or imipramine.

Semi-structured interviews revealed differences between psychiatrists regarding the position of TCAs in treatment of MDD and dose-adjustment. Hospital psychiatrists were the most inclined to prescribe TCAs and private practice holders the least, possibly because of a difference in patient populations and ease of monitoring the patient. Many psychiatrists experienced barriers in TCA dose-adjustment, although significantly less for nortriptyline compared to other TCAs. Important barriers were adverse effects hindering dose up-titration, managerial problems related to TDM and usual maintenance doses not corresponding with therapeutic plasma concentrations.

### Guideline adherence

A comparison between the Dutch guideline [10, 18] and clinical practice, i.e. information provided by health records and interviews, showed that the guideline was not consistently followed. Despite the guideline presenting TCAs as first-line treatment option, all psychiatrists selected TCAs as second- or third-line antidepressants, also in hospitalized patients. Health records and interviews demonstrated that TDM is generally applied within the first weeks of treatment to guide dose-adjustment, whereas the guideline advises TDM only in case of non-response [10] or in elderly patients with severe MDD [18].

Several factors promoted TCA selection in clinical practice but were not consistently mentioned in the guidelines. For instance, severe depression promoted selection of TCAs by psychiatrists in line with several studies suggesting that TCAs are more effective than SSRIs in treatment of severe melancholic depression [35–37]. However, only the WFSBP guideline considered depression severity explicitly, presenting TCAs as first-line antidepressants for severe but not for milder depression [17]. In accordance with most selected guidelines, the interviews with psychiatrists suggested that nortriptyline was the preferred TCA in elderly patients and clomipramine in patients with comorbid anxiety disorders, although the latter could not be confirmed by our analysis of health records, possibly due to a limited number of patients with comorbid anxiety.

The Dutch guideline did not consider pharmacogenetics, which may be explained by its publication date (2013) [10]. Nowadays, several genotype-informed dosing directions are available, but these are not embedded in general antidepressant prescribing guidelines [8, 38]. The interviews suggested that separate guidelines for different aspects of TCA dosing, e.g., pharmacogenetics and TDM, may promote heterogeneity in clinical practice.

### Strengths and limitations

An important strength of this study is that different methods were combined. Health records were selected randomly, divided over five centers in the Netherlands. Different types of centers were included. Moreover, the interviewed psychiatrists were carefully selected to represent Dutch psychiatrists. A limitation is that we reviewed a relatively small number of guidelines and health records, although representative for the Netherlands. Also, we interviewed only psychiatrists who were used prescribing TCAs. Psychiatrists prescribing TCAs less often and other health care professionals may perceive different promotors and barriers influencing TCA prescription.

## Conclusions

Today, there is considerable variation in guidelines and clinical practice regarding TCA prescription for MDD. This study confirms that TCAs are generally prescribed as a later step in the pharmacological treatment of MDD, but also that the present-day position of TCAs in treatment of MDD and optimal dosing strategies require further elaboration. In clinical practice, the choice for TCAs was often motivated by guideline directions but was also strongly guided by preferences of patients and psychiatrists, based on a variety of reasons. Health records showed that TDM was frequently applied and that most patients attained therapeutic plasma concentrations within the first two months of treatment. Yet, many psychiatrists perceived barriers in dose-adjustment, although clearly less for nortriptyline compared to other TCAs. To promote consistency in clinical practice we recommend development of an up-to-date guidance integrating TCA selection and dosing, including the roles of TDM and pharmacogenetics, if possible at a European level. Such a guideline is currently lacking and would contribute to optimized TCA prescription, whereby efficacy, safety and tolerability may be increased.

## Supplementary Information

**Additional file 1: Supplement 1.** Interview guide as used in semi-structured interviews with psychiatrists.

## Data Availability

The datasets used and analyzed during the current study are available from the corresponding author on reasonable request.

## Abbreviations

APA: American psychiatric association
CANMAT: Canadian network for mood and anxiety treatments
CYP2D6: Cytochrome P-450 2D6
CYP2C19: Cytochrome P-450 2C19
DGPPN: Deutsche Gesellschaft für Psychiatrie Psychotherapie und Nervenheilkunde
MDD: Major depressive disorder
MAO inhibitors: Monoamine oxidase inhibitors
NICE: National Institute for health and care excellence
OCD: Obsessive compulsive disorder
SmPC: Summary of product characteristics
SNRI: Serotonin-noradrenaline reuptake inhibitor
SSRI: Selective serotonin reuptake inhibitor
TDM: Therapeutic drug monitoring
TCA: Tricyclic antidepressant
WFSBP: World federation of societies of biological psychiatry
WHO: World health organization
