# Supplementary material for: Tricyclic antidepressants for major depressive disorder: a comprehensive evaluation of current practice in the Netherlands
Source: BMC Psychiatry. 2021 Oct 1;21:481. doi: 10.1186/s12888-021-03490-x (PMC8487125; doi:10.1186/s12888-021-03490-x)
Supplement: Supplementary file 1 — Additional file 1: Supplement 1. Interview guide as used in semi-structured interviews with psychiatrists. [file 12888_2021_3490_MOESM1_ESM.docx]

Supplement 1. Interview guide as used in semi-structured interviews with psychiatrists

| **Part 1**  **Choice for Tricyclic Antidepressants** | Open questions | What are your general experiences prescribing tricyclic antidepressants for major depression?  When do you consider tricyclic antidepressants for major depression?  For what type patients do you consider tricyclic antidepressants?  For what reasons do you choose for a tricyclic antidepressant, if you do so?  For what reasons do you choose for other antidepressants, if you do so?  What are your considerations in choosing between nortriptyline, clomipramine, imipramine or amitriptyline?  What are your experiences prescribing nortriptyline, clomipramine, imipramine and amitriptyline? |
| --- | --- | --- |
|  | Closed questions | Do the following factors influence your choice for a tricyclic antidepressant: depression severity, age, comedications, comorbidities, clinical setting, effect of previous treatment, use of previous antidepressants? If yes, how? If not, why?  Do you use protocols or guidelines in choosing antidepressants? If yes, what protocols or guidelines do you use? |
| **Part 2**  **Dosing of Tricyclic Antidepressants** | Open questions | Could you describe your typical dosing strategy for nortriptyline, clomipramine, imipramine and amitriptyline?  What is an optimal dosing strategy for tricyclic antidepressants according to you?  Could there be reasons to prescribe higher or lower doses? If yes, what reasons?  What is the role of therapeutic drug monitoring in dosing of tricyclic antidepressants in your own practice?  What is the role of other personalized dosing strategies, such as pharmacogenetics, in your own practice? |
|  | Closed questions | Do the following factors influence dosing of tricyclic antidepressants: depression severity, age, comedications, comorbidities, clinical setting, effect of previous antidepressant treatment? If yes, how? If not, why?  Do you use protocol or guidelines in dosing of tricyclic antidepressants? If yes, what protocols or guidelines do you use? |

Semi-structured interviews were based on this interview guide but questions were influenced by the answers given.

The interview was divided in two parts. In the first part psychiatrists were questioned about how they choose an

antidepressant. In the second part psychiatrists were questioned about dosing tricyclic antidepressants.
